# Leading patterns of internet-addicted behavior in adolescents in central siberia according to the results of the CIAS test

**DOI:** 10.1192/j.eurpsy.2021.1545

**Published:** 2021-08-13

**Authors:** N. Semenova, S. Tereshchenko, L. Evert L, Y. Kostyuchenko, M. Shubina

**Affiliations:** 1 Scientific Research Institute For Medical Problems Of The North, Federal Budgetary Scientific Institution «Federal Research Centre «Krasnoyarsk Scientific Centre of Siberian Division of Russian Academy of Sciences», Krasnoyarsk, Russian Federation; 2 Research Institute For Medical Problems In The North, Federal Research Center “Krasnoyarsk Scientific Center of the Siberian Branch of the RAS”, Krasnoyarsk, Russian Federation

**Keywords:** Internet, Addiction, patterns, adolescents

## Abstract

**Introduction:**

Knowing the leading patterns will help timely predict that addictive behavior is being formed.

**Objectives:**

To identify the leading patterns of addictive behavior in adolescents in Central Siberia according to the results of the CIAS test.

**Methods:**

200 adolescents aged 11-18, 69 males and 131 females, with Internet addiction living in an urban area (Krasnoyarsk) were surveyed. The patterns of addictive behavior were assessed using the CIAS test, which includes scales of key symptoms: “Com” (compulsive symptoms), “Wit” (withdrawal symptoms), “Tol” (tolerance symptoms); and negative consequences scales: “IH” (intrapersonal and health problems), “TM” (problems with time management).

**Results:**

The mean results (M) of key symptoms were obtained at 14.56 on the “Com” scale, 15.27 on the “Wit” scale, 12.23 on the “Tol”. The mean indices of negative manifestations were obtained at 17.00 on the “IH” scale and 13.94 on the “TM” scale. When comparing the mean results of the scales of addicted behavior by the method of one-way analysis of variance (ANOVA), statistically significant differences between representatives of different sex and age groups were not revealed (p> 0.05).

**Conclusions:**

The leading key symptoms of Internet-addicted behavior in Central Siberia adolescents include withdrawal symptoms: decreased mood, anxiety and irritation in the absence of access to the Internet. Symptoms of negative consequences include decreased social contacts, reduced communication with family members, and problems at school. When such symptoms emerge, one should suspect the formation of Internet addiction and carry out the necessary diagnostics for timely intervention. The study was funded by RFBR project № 18-29-22032\18.

**Conflict of interest:**

The study was funded by RFBR project № 18-29-22032\18.

